# 

**Published:** 1911-12

**Authors:** 


### CONTENTS.

*Page*

The Present Position of the Surgery of Malignant Growths. By CHARLES A. MORTON, F.R.C.S. ... ... ... ... ... ... 289

Household Syphilis (Syphilis Œconomica), with observations on acquired syphilis in infants. By J. A. NIXON, B.A., M.B. Cantab, F.R.C.P. ... ... ... ... ... ... ... ... ... 301

Some observations on Tea-poisoning. By NEWMAN NEILD, M.B. Vict., M.R.C.P. ... ... ... ... ... ... ... 312

Cardiac Arrhythmia: five cases. By CAREY COOMBS, M.D. Lond., M.R.C.P. ... ... ... ... ... ... ... ... ... 321

Herpes and Brachial Neuritis. By G. MUNRO SMITH, M.R.C.S. ... 332

The Bristol Medical Library. By L. M. GRIFFITHS, M.R.C.S. Eng., L.R.C.P. Edin. ... ... ... ... ... ... ... ... .. 335

Progress of the Medical Sciences:

Medicine. J. R. CHARLES, M.A., M.D. Cantab., M.R.C.P. ... 345

Surgery. HAROLD F. MOLE, F.R.C.S. ... ... ... ... ... 348

Reviews of Books ... ... ... ... ... ... ... ... 353

Editorial Notes ... ... ... ... ... ... ... ... ... 366

Notes on Preparations for the Sick ... ... ... ... ... 373

The Library of the Bristol Medico-Chirurgical Society ... ... 376

Meetings of Societies ... ... ... ... ... ... ... ... 378

Obituary Notices ... ... ... ... ... ... ... ... ... 382

Local Medical Notes ... ... ... ... ... ... ... ... 383

